# Supplementary material for: Glucose Concentration in Cell Culture Medium Influences the BRCA1-Mediated Regulation of the Lipogenic Action of IGF-I in Breast Cancer Cells
Source: Int J Mol Sci. 2020 Nov 17;21(22):8674. doi: 10.3390/ijms21228674 (PMC7698585; doi:10.3390/ijms21228674)
Supplement: Supplementary file 1 [file ijms-21-08674-s001.zip › ijms-981629-supplementary.docx]

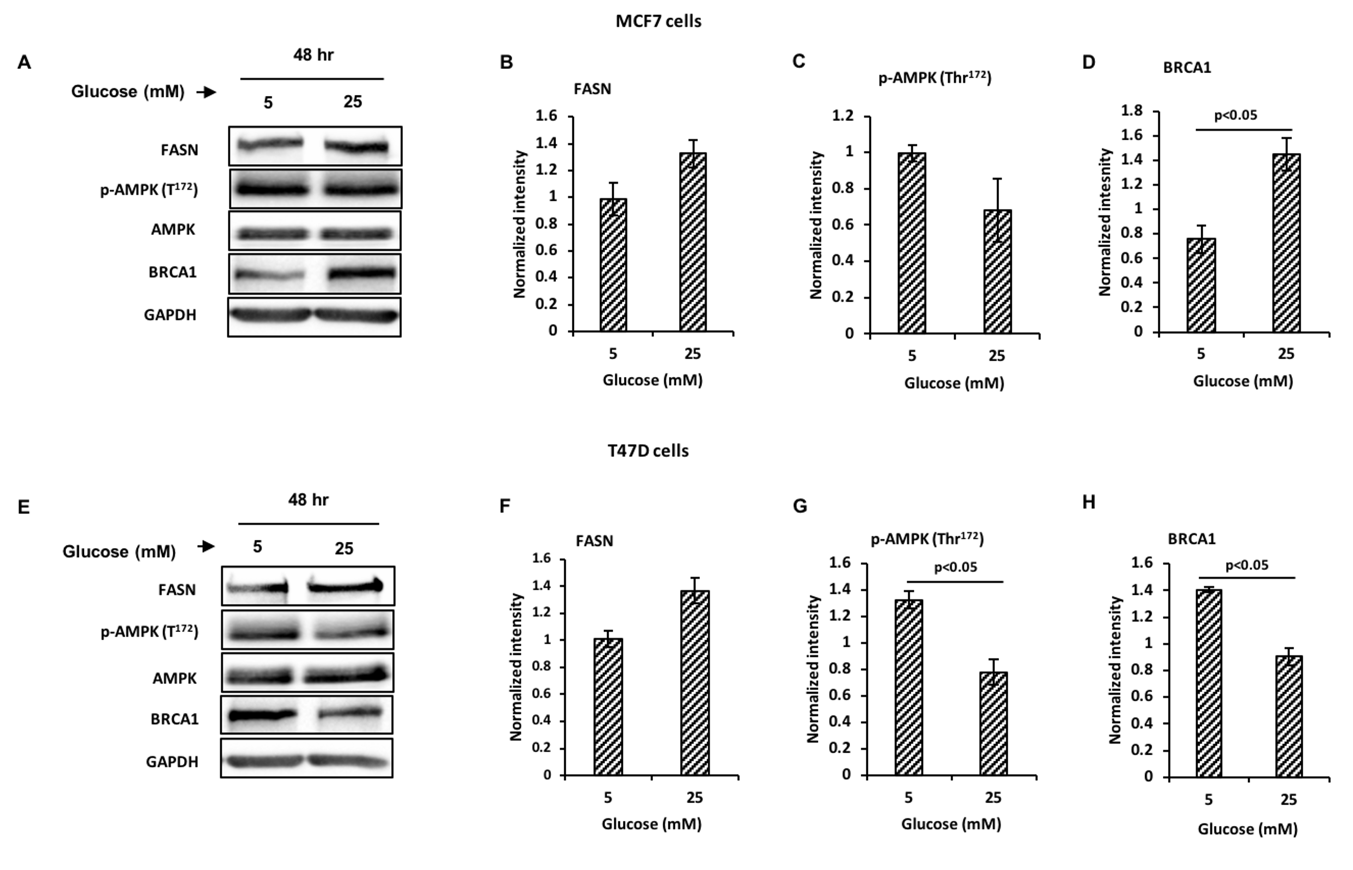


**Figure S1.** Regulation of key lipogenic proteins by glucose in MCF7 and T47D breast cancer cells. (**A**–**D**) MCF7 and (**E**–**H**) T47D were cultured in normal and high glucose in serum-free media for 48 h. Lysates were analysed by western blotting and representative immunoblots of FASN, AMPK and BRCA1 are shown. Graphs represent densitometry analysis of (**B**,**F**) FASN normalized to GAPDH, (**C**,**G**) p-AMPK (Thr^172^) normalized to total AMPK and (**D**,**H**) BRCA1 normalized to GAPDH. All results shown are representative of three independent experiments and the graphs represent mean ± SEM. Independent samples *t*-test was used to compare means of 2 groups.
